# Supplementary material for: Non-Poissonian photon statistics from macroscopic photon cutting materials
Source: Nat Commun. 2017 May 24;8:15537. doi: 10.1038/ncomms15537 (PMC5458076; doi:10.1038/ncomms15537)
Supplement: Supplementary Information — Supplementary Figures, Supplementary Notes and Supplementary References [file ncomms15537-s1.pdf]

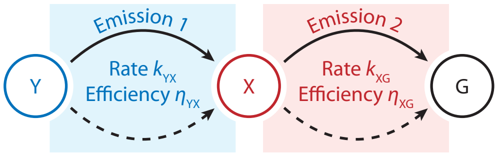

Supplementary Figure 1 | Simplified model of a photon-cutting material. A photon cutting material decays from a high-energy excited state Y (blue circle) via an intermediate state X (red circle) to the ground state G (black circle). The two steps are shaded blue and red, respectively. Both steps can, in general, be radiative (solid arrows) or non-radiative (dashed arrows), resulting in total decay rates k_{YX} and k_{XG} and emission efficiencies η_{YX} and η_{XG}.

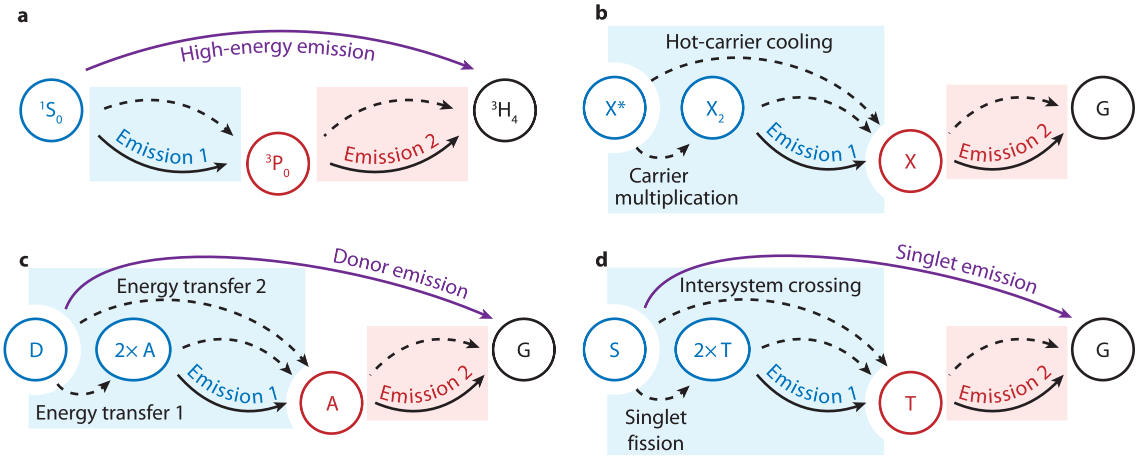

Supplementary Figure 2 | Different types of photon-cutting materials. The excited-state dynamics of various photon-cutting materials. The highest excited state(s) are shown as blue circles, the intermediate state in red, and the ground state in black. Radiative steps are depicted with solid arrows, non-radiative steps with dashed arrows. Blue and red shaded areas highlight the two steps in the photon-cutting process. The magnitude of the bunching signal depends on the efficiencies of these steps. **(a)** The lanthanide-doped phosphor $\mathrm{NaLaF_4{:}Pr^{3+}}$, studied in detail in the main text, is an example of a photon cutter by means of cascade emission. The photon-cutting process takes place on a single luminescent centre, here $\mathrm{Pr^{3+}}$, that emits its excited-state energy in two separate steps. **(b)** Carrier multiplication from a hot-exciton state in a quantum dot generates a biexciton state. This state decays to the quantum dot ground state in a cascade process, via the single-exciton state as intermediate. Hot-carrier cooling is a non-radiative pathway from the hot-exciton state competing with carrier multiplication. **(c)** In an important class of photon cutters, an excited donor centre (D) distributes its energy over two acceptor centres (A). Direct (radiative) decay of the donor to its ground state may compete with energy transfer to the acceptors. In addition, an energy-transfer pathway that excites only a single acceptor may exist. **(d)** Singlet fission in organic dyes is a form of photon cutting by energy distribution over two centres (as in panel c). The 'donor' is the singlet state (S), the 'acceptors' are triplet states (T).

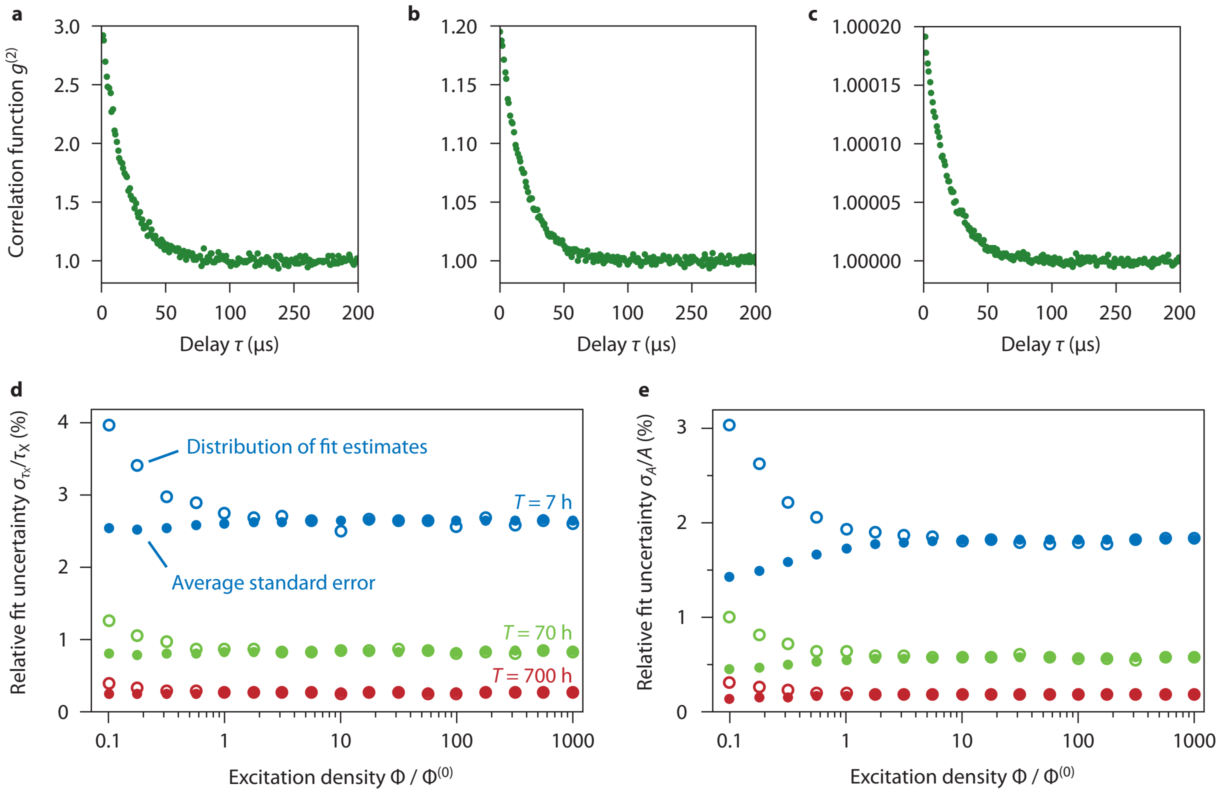

Supplementary Figure 3 | The effect of excitation density on bunching amplitude and noise. (a-c) Simulated cross-correlation functions for photon-cutting emission by $NaLaF_4$:Pr^{3+}, **(b)** for the experimental parameters as in Fig. 2 of the main text, **(a)** for 10× weaker excitation, and **(c)** for 1000× stronger excitation. The bunching amplitude and noise both scale inversely with excitation power, so that the three correlation functions can be distinguished only from the range depicted on the y-axis. **(d,e)** From a statistical analysis of 1000 simulated experiments per excitation density, we calculate how accurately we can extract **(d)** the excited-state lifetime and **(e)** the bunching amplitude from a weighted least-squares fit. We consider a range of four orders of magnitude in excitation density normalised to the density used in Fig. 2 of the main text. Open circles depict the variation of fitted values from 1000 simulations (as one standard deviation), filled circles the average fit uncertainty (as one standard error) of the 1000 fits. The different colors represent different experiment durations of $T = 7$ h (blue), $T = 70$ h (green; as in Fig. 2 of the main text), and $T = 700$ h (red). While longer experiments allow for more accurate estimates of the excited-state lifetime and bunching amplitude, the excitation density has only limited effect except at very low densities. For such low densities the uncertainty is slightly increased. This is the result of the assumption of Gaussian noise in the weighted least-squares fit procedure used, and could be potentially be avoided by using more advanced models for the noise [7].

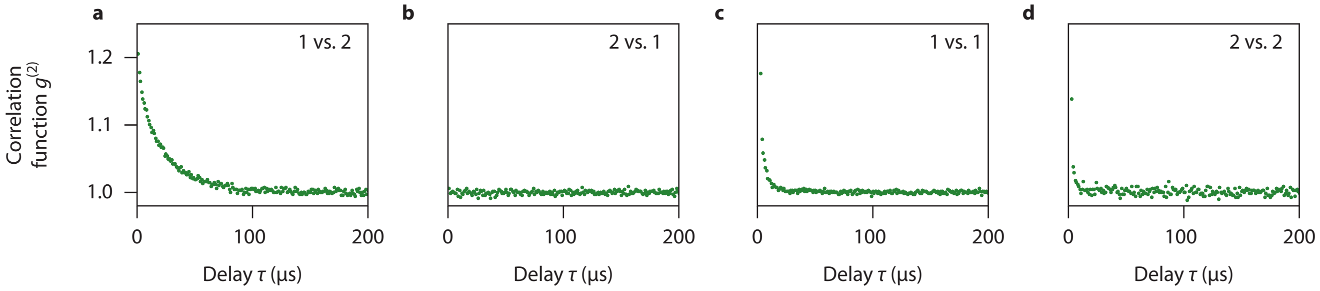

Supplementary Figure 4 | Four correlation functions $g^{(2)}$ for photon detection traces on two detectors. Correlation functions for the experiment presented in Fig. 2f on the main text, in which the cascade emission is separated spectrally over two detectors. **(a,b)** The cross-correlation of the signals on the two detectors (**(a)** 1 vs. 2 and **(b)** 2 vs. 1) shows that blue photons on detector 1 are followed by red-green photons on detector 2. **(c,d)** However, there is also a bunching signal observed in the auto-correlation functions of the separate detector signals (**(c)** 1 vs. 1 and **(d)** 2 vs. 2). This is caused by afterpulsing.

SUPPLEMENTARY NOTE 1: PHOTON BUNCHING FROM VARIOUS TYPES OF PHOTON-CUTTING MATERIALS

Supplementary Fig. 1 shows a simple model of a photon-cutting material. The material decays from a high-energy excited state Y (blue circle) in two steps. In the first step (shaded blue) it decays to intermediate excited state X (red circle), and in the second step (shaded red) to ground state G (black circle). The two steps can be made radiatively (solid arrows), or non-radiatively (dashed arrows). These two possibilities result in a total decay rate k_{YX} and an emission efficiency η_{YX} for state Y, and k_{XG} and η_{XG} for state X.

In the main text we consider the photon cutter $\mathrm{NaLaF_4{:}Pr^{3+}}$, which is a near-ideal material for our bunching experiments: the two photons emitted in a cascade process can be spectrally distinguished, and the photon emission efficiency of both steps is high enough that detector dark counts can be neglected. The experimental cross-correlation function therefore matches the simplified expression

$$g^{(2)}(\tau) = 1 + \frac{1}{\bar{N}_{\mathrm{X}}} \mathrm{e}^{-k_{\mathrm{XG}}\tau} \tag{1}$$

derived in the Methods section. We call this most ideal case 'scenario A'. In this section we consider different scenarios of increasing complexity:

- scenario B: the cut photons cannot be spectrally distinguished;
- scenario C: spectral distinction is possible, but detector dark counts are non-negligible;
- scenario D: spectral distinction is not possible, and detector dark counts cannot be neglected.

In each case, the energy level structure and dynamics of the N optical centres in the system are as in Supplementary Fig. 1. This structure results in steady-state populations of $\bar{N}_{\mathrm{Y}} = \Phi N/k_{\mathrm{YX}}$ and $\bar{N}_{\mathrm{X}} = \Phi N/k_{\mathrm{XG}}$ of states Y and X, respectively, where Φ is the excitation rate from state G to state Y.

Scenario B: photon bunching without spectral separation

For scenario B, we assume that the two photon-emission steps in the cascade are not spectrally separated, but that all photons emitted are sent through a 50/50 beam splitter and arrive at either of two detectors with equal probability. This is the situation of the experiment of Fig. 3d in main text. The average count rate is the same for the two detectors, and depends on the steady-state populations of both excited states:

$$\langle I_{1,2}(t)\rangle = \eta \left(\eta_{\mathrm{YX}} k_{\mathrm{YX}} \bar{N}_{\mathrm{Y}} + \eta_{\mathrm{XG}} k_{\mathrm{XG}} \bar{N}_{\mathrm{X}}\right)/2, \tag{2}$$

where η is the collection and detection efficiency of the optical setup, and the factor 2 accounts for the effect of the 50/50 beam splitter. A click on detector 1 now has a finite probability of

$$p = \frac{\eta_{\mathrm{YX}} k_{\mathrm{YX}} \bar{N}_{\mathrm{Y}}}{\eta_{\mathrm{YX}} k_{\mathrm{YX}} \bar{N}_{\mathrm{Y}} + \eta_{\mathrm{XG}} k_{\mathrm{XG}} \bar{N}_{\mathrm{X}}} = \frac{\eta_{\mathrm{YX}}}{\eta_{\mathrm{YX}} + \eta_{\mathrm{XG}}} \tag{3}$$

to originate from a photon emitted in the transition Y $\to$ X. Consequently, the expectation value $\langle N_{\mathrm{X}}(\tau)\rangle$ for the population of state X at delay time τ after a click on detector 1 is increased with respect to the steady-state value:

$$\langle N_{\mathrm{X}}(\tau)\rangle = \bar{N}_{\mathrm{X}} + p\,\mathrm{e}^{-k_{\mathrm{XG}}\tau} \tag{4}$$

And the cross-correlation function $g^{(2)}$ can be expressed as

$$\begin{aligned} g^{(2)}(\tau) &= \frac{\langle I_1(t) I_2(t+\tau)\rangle}{\langle I_1(t)\rangle\langle I_2(t)\rangle} = 1 + \frac{\eta\eta_{\mathrm{XG}} k_{\mathrm{XG}}/2}{\langle I_2(t)\rangle} p\,\mathrm{e}^{-k_{\mathrm{XG}}\tau} \\ &= 1 + \frac{\eta_{\mathrm{YX}}\eta_{\mathrm{XG}}}{(\eta_{\mathrm{YX}} + \eta_{\mathrm{XG}})^2} \frac{1}{\bar{N}_{\mathrm{X}}} \mathrm{e}^{-k_{\mathrm{XG}}\tau}. \end{aligned} \tag{5}$$

Since the two detectors in this scenario have equal probabilities to detect either photon of the cascade, the bunching symmetric with respect to $\tau = 0$. Negative values of τ are for photon pairs of which the first arrived at detector 2.

Scenario C: photon bunching with spectral separation, and with dark counts

In scenario C the photons emitted in the cascade process are spectrally distinct and are sent to separate detectors. Detector 1 collects emission from the first transition Y $\to$ X, detector 2 from the second transition X $\to$ G. In contrast to the experiments in the main text, however, the detector dark counts cannot be neglected. We assume that the two detectors have equal dark

count rates D, and equal detection efficiencies for the photons directed to them (combined with the collection efficiency of the optical setup into an overall detection and collection efficiency η). The average count rates are then:

$$\langle I_1(t)\rangle = \eta\eta_{\mathrm{YX}}k_{\mathrm{YX}}\bar{N}_{\mathrm{Y}} + D \tag{6}$$
$$\langle I_2(t)\rangle = \eta\eta_{\mathrm{XG}}k_{\mathrm{XG}}\bar{N}_{\mathrm{X}} + D \tag{7}$$

A click on detector 1 has a probability of

$$p = \frac{\eta\eta_{\mathrm{YX}}k_{\mathrm{YX}}\bar{N}_{\mathrm{Y}}}{\eta\eta_{\mathrm{YX}}k_{\mathrm{YX}}\bar{N}_{\mathrm{Y}} + D} \tag{8}$$

to be due to a photon emitted in a transition Y → X. As before, the expectation value for the population of state X following a click on detector 1 is affected by the finite value of p (see equation (4)). The cross-correlation function $g^{(2)}$ is given by

$$\begin{aligned} g^{(2)}(\tau) &= \frac{\langle I_1(t)I_2(t+\tau)\rangle}{\langle I_1(t)\rangle\langle I_2(t)\rangle} = 1 + \frac{\eta\eta_{\mathrm{XG}}k_{\mathrm{XG}}}{\langle I_2(t)\rangle}p\,\mathrm{e}^{-k_{\mathrm{XG}}\tau} \\ &= 1 + \frac{\eta^2\eta_{\mathrm{YX}}\eta_{\mathrm{XG}}\Phi N k_{\mathrm{XG}}}{(\eta\eta_{\mathrm{YX}}\Phi N + D)\,(\eta\eta_{\mathrm{XG}}\Phi N + D)}\mathrm{e}^{-k_{\mathrm{XG}}\tau}. \end{aligned} \tag{9}$$

In contrast to scenarios A and B, the cross-correlation function for scenario C depends explicitly on the collection and detection efficiency η. In the previous scenarios, a finite value for η merely reduced the total signal. In scenario C, however, Poissonian dark counts cause a flat background on $g^{(2)}$ which becomes more prominent as the value of η decreases. As expected, equation (9) reduces to equation (1) in the limit of $D \to 0$.

Scenario D: photon bunching without spectral separation, but with dark counts

Finally, we consider scenario D where photons emitted in the cascade are not spectrally separated (as in scenario B), and the detector dark counts are non-negligible (as in scenario C). The two detectors have equal average count rates:

$$\langle I_{1,2}(t)\rangle = \eta\left(\eta_{\mathrm{YX}}k_{\mathrm{YX}}\bar{N}_{\mathrm{Y}} + \eta_{\mathrm{XG}}k_{\mathrm{XG}}\bar{N}_{\mathrm{X}}\right)/2 + D. \tag{10}$$

The probability that a click on detector 1 originates from a photon emitted in a transition Y → X is given by

$$p = \frac{\eta\eta_{\mathrm{YX}}k_{\mathrm{YX}}\bar{N}_{\mathrm{Y}}/2}{\eta\left(\eta_{\mathrm{YX}}k_{\mathrm{YX}}\bar{N}_{\mathrm{Y}} + \eta_{\mathrm{XG}}k_{\mathrm{XG}}\bar{N}_{\mathrm{X}}\right)/2 + D}. \tag{11}$$

Knowing the expectation value for the population of state X after a click on detector 1 (equation (4)), we find the following expression for the normalised cross-correlation function:

$$\begin{aligned} g^{(2)}(\tau) &= \frac{\langle I_1(t)I_2(t+\tau)\rangle}{\langle I_1(t)\rangle\langle I_2(t)\rangle} = 1 + \frac{\eta\eta_{\mathrm{XG}}k_{\mathrm{XG}}/2}{\langle I_2(t)\rangle}p\,\mathrm{e}^{-k_{\mathrm{XG}}\tau} \\ &= 1 + \frac{\eta^2\eta_{\mathrm{YX}}\eta_{\mathrm{XG}}\Phi N k_{\mathrm{XG}}/4}{\left[\eta\Phi N\left(\eta_{\mathrm{YX}} + \eta_{\mathrm{XG}}\right)/2 + D\right]^2}\mathrm{e}^{-k_{\mathrm{XG}}\tau}. \end{aligned} \tag{12}$$

As in scenario C, the cross-correlation depends explicitly on the collection and detection efficiency η. Equation (5) is obtained from equation (12) by taking the limit $D \to 0$.

SUPPLEMENTARY NOTE 2: VARIOUS PHOTON CUTTING MATERIALS

Cascade emission from lanthanide ions

The lanthanide-doped phosphor $NaLaF_4$:Pr^{3+}, studied in detail in the main text, is an example of a photon cutter by means of cascade emission.[1] The photon-cutting process takes place on a single luminescent centre, here Pr^{3+}, that emits its excited-state energy in two separate steps. This system can be mapped on the simplified model (Supplementary Fig. 1) and the corresponding equations (see above), as we show in Supplementary Fig. 2a.

The highly excited state $^1S_0$ is identified with state Y in the model, and the intermediate state $^3P_0$ with state X. To be very precise, state $^1S_0$ decays to state $^1I_6$ (giving rise to the emission line at 406 nm with a radiative efficiency of 100 % [1]; see Fig. 2c in the main text) which then very rapidly relaxes to the $^3P_0$ state. We do not have to consider these sequential substeps separately, because the second substep follows the first on a very fast timescale and with a near-unity probability. The second step in the photon-cutting process is relaxation from the $^3P_0$ state (= X) to the ground states $^3H_6$ (giving rise to emission at 480 nm; see Fig. 2c in the main text) and $^3H_4$ (607 nm). Since these resulting emissions come from the same excited state, and are not spectrally separated in our experiment, we can consider that the $^3H_6$ and $^3H_4$ states together constitute the ground

state G. The radiative efficiency of the second step is 40 % for a Pr^{3+} concentration of 1 %, which is reduced at higher Pr^{3+} concentrations because of efficient cross-relaxation.[1]

In addition to the decay processes from Y to X and from X to G, as in the simplified model of Supplementary Fig. 1, decay from the highest-energy excited state $^1S_0$ (= Y) of Pr^{3+} can bypass the intermediate level $^3P_0$ (= X). Such decay pathways result in high-energy photons with wavelengths of 200–350 nm (see Ref. [1]). In our experiment, we filter out these ultraviolet photons with a band-pass filter after the objective (see Fig. 2b in the main text). Pr^{3+} ions bypassing the intermediate state are therefore invisible to our experiment. These ions cannot be distinguished from ions that remained in the ground state. The effect of high-energy emission (purple arrow in Supplementary Fig. 2a) on our bunching experiment can therefore be taken into account by considering an 'effective' excitation rate $\Phi \to \phi\Phi$, where ϕ is the probability that the high excited state Y (= $^1S_0$) decays via the cascade pathway Y → X → G.

Multi-exciton generation in semiconductor nanocrystals

Multi-exciton generation (or carrier multiplication) in semiconductor nanocrystals can result in cascade emission. A high-energy hot-carrier state X^* decays into a biexciton state X_2. For applications in photovoltaics using quantum dot solids, this biexciton state should then separate into four charge carriers. In individual nanocrystals, however, the biexciton state decays in a two-step process (see Supplementary Fig. 2b): first from the biexciton state X_2 to the exciton state X, and then further to the ground state G. Both steps can be radiative (solid arrows) or non-radiative (dashed arrows). The photons emitted in the two steps are typically so close in energy that spectral separation would not be possible (scenarios B and D above). In addition, the hot-carrier state X^* can decay to the exciton state X directly and non-radiatively, namely through hot-carrier cooling (dashed arrow).

The hot-carrier state X^* in multi-exciton generation can be identified with high-energy excited state Y in our model of Supplementary Fig. 1, and the exciton state X with the intermediate level X. Three decay pathways exist from state Y to state X: (1) non-radiative hot-carrier cooling, (2) carrier multiplication + non-radiative decay of the X_2 state, and (3) carrier multiplication + radiative decay of the X_2 state. The efficiency η_{YX} of the first step in our model would correspond to the efficiency of the pathway 3 in the competition between these three pathways. A photon-bunching experiment could identify whether multi-exciton generation occurs, and would provide indirect information about the relative efficiencies of carrier multiplication and hot-carrier cooling.

Distribution of energy over two optical centres

A third class of photon-cutting materials are those in which an optical centre is initially excited to a high-energy state, after which this energy is distributed over two separate centres. This distribution process can take many forms. For example, a singlet excitation in a film of pentacene molecules can decay into two triplet excitations in neighbouring molecules.[2] While such triplet states themselves may be hardly luminescent, the triplet-state energy can eventually be released as (bunched) photons via an energy transfer step to bright semiconductor nanocrystals.[3, 4] Distribution of excited-state energy has also been reported in silicon quantum dot ensembles. Hot-carrier energy in one of these quantum dots can be transferred to a second quantum dot in the vicinity, resulting in two separate excitations.[5] As a third example, ensembles of lanthanide ions can exhibit energy transfer processes that distribute a high-energy excitation over two acceptors. An example of this occurs in YPO_4 doped with Tb^{3+} and Yb^{3+}, where a cooperative energy-transfer process can distribution the excited-state energy from one Tb^{3+} ion in the $^5D_4$ state to two Yb^{3+} ions.[6]

In Supplementary Fig. 2c we depict a general system where one optical donor centre can distribute its energy over two acceptor centres, and in Supplementary Fig. 2d for the more specific case of singlet fission. The high-energy state Y in our model (Supplementary Fig. 1) can be identified with the excited donor (D), or with the singlet state (S). The intermediate state X in our model is a single excited acceptor (A), or a single excited triplet state (T). In addition to the desired energy-transfer pathway that excites two acceptor centres (labeled 'energy transfer 1' in panel c, and 'singlet fission' in panel d), there may be other pathways that yield only one excited acceptor centre (labeled 'energy transfer 2' in panel c, and 'intersystem crossing' in panel d). In addition, the high-energy excited state could have a (radiative) pathway that bypasses the intermediate state (purple arrows). Finally, the two decay steps (from two acceptors/triplets to one, and from one to zero) might be radiative or non-radiative.

In systems as described here, the emissions 1 and 2 can typically not be separated spectrally. However, they can be separated from high-energy emissions that bypass the intermediate state. This would mean for example that an experiment on YPO_4:Tb^{3+},Yb^{3+} would collect infrared (cut) photons emitted by Yb^{3+} but not high-energy photons emitted by Tb^{3+}. We assume here that the experiment is set up in this way. The efficiency η_{YX} of the first step in our model then corresponds to the efficiency radiative pathway from Y to X, compared to all non-radiative pathways that end in intermediate state X (i.e. all pathways in the blue-shaded areas in Supplementary Fig. 2c,d). The photon-bunching experiment could therefore identify

whether the desired energy transfer step ('energy transfer 1' or 'singlet fission') occurs, and provide information about the efficiency.

SUPPLEMENTARY NOTE 3: ANALYSIS OF EXPERIMENTAL BUNCHING DATA

To obtain the cross-correlation function from the discrete experimental data and Monte Carlo simulations, we calculate time differences τ between all pairs of photons recorded at discrete times t_1 on detector 1 and t_2 on detector 2. The cross-correlation function $g^{(2)}$ is a histogram of the time differences, binned at a bin width $\delta\tau$. We normalise the cross-correlation function by multiplying with $(I_1 I_2 T \delta\tau)^{-1}$, where $I_{1,2}$ are the average intensities on detectors 1 and 2. To obtain the bunching amplitude and its confidence interval, we fit equation (1) to the cross-correlation function. In Supplementary Figure 3 we study the uncertainty in the fitted parameters.

In Fig. 3f we plot the amplitude of the noise in the cross-correlation function as a function of T. The noise σ is the root-mean-square deviation of the measured correlation function at negative delay times $\tau < 0$ from a value of 1:

$$\sigma = \sqrt{\frac{1}{N_i}\sum_i^{N_i}\left(g_i^{(2)} - 1\right)^2} \tag{13}$$

where $g_i^{(2)}$ is the normalised cross-correlation in time bin i, and the sum $\sum_i$ runs over all data points at $\tau < 0$.

Using that $\sigma = (I_1 I_2 T \delta\tau)^{-1/2}$ we can derive the expected noise in the scenarios A–D. For scenario A this is

$$\sigma = (\eta_{\mathrm{YX}}\eta_{\mathrm{XG}} T\delta\tau)^{-1/2}(k_{\mathrm{XG}}\eta\bar{N}_{\mathrm{X}})^{-1}. \tag{14}$$

For the bunching peak to be visible over the noise the on the cross-correlation function, the amplitude (which for scenario A equals $\bar{N}_{\mathrm{X}}^{-1}$; see equation (1)) must be sufficiently high:

$$\bar{N}_{\mathrm{X}}^{-1} > (\eta_{\mathrm{YX}}\eta_{\mathrm{XG}} T\delta\tau)^{-1/2}(k_{\mathrm{XG}}\eta\bar{N}_{\mathrm{X}})^{-1} \tag{15}$$

To satisfy this condition, the experiment duration T must be

$$T > \frac{1}{k_{\mathrm{XG}}^2\eta^2\eta_{\mathrm{YX}}\eta_{\mathrm{XG}}\delta\tau}. \tag{16}$$

In other words, with ideal detectors photon bunching should be observable from an ensemble of optical centres irrespective of the number of centres investigated simultaneously and irrespective of the excitation rate, as long as the experiment lasts sufficiently long.

For scenario B the expected noise on the cross-correlation function is

$$\sigma = (T\delta\tau)^{-1/2}(\eta\Phi N\left(\eta_{\mathrm{YX}} + \eta_{\mathrm{XG}}\right)/2)^{-1} \tag{17}$$

and the bunching would be visible over the noise if

$$T > \frac{4}{\delta\tau}\left(\frac{\eta_{\mathrm{YX}} + \eta_{\mathrm{XG}}}{\eta\eta_{\mathrm{YX}}\eta_{\mathrm{XG}}k_{\mathrm{XG}}}\right)^2 \tag{18}$$

Hence, in the most ideal case ($\eta_{\mathrm{YX}}, \eta_{\mathrm{XG}} = 1$), the clear observation of bunching requires longer measurements by a factor 16 in the absence of spectral separation (scenario B) compared to scenario A with spectral separation.

For scenario C the expected noise is

$$\sigma = (T\delta\tau)^{-1/2}\left(\eta\eta_{\mathrm{YX}}\Phi N + D\right)^{-1/2}\left(\eta\eta_{\mathrm{XG}}\Phi N + D\right)^{-1/2} \tag{19}$$

and the threshold for the bunching amplitude to be higher than the noise is

$$T > \frac{(\eta\eta_{\mathrm{YX}}\Phi N + D)(\eta\eta_{\mathrm{XG}}\Phi N + D)}{\eta^4\eta_{\mathrm{YX}}^2\eta_{\mathrm{XG}}^2\Phi^2N^2k_{\mathrm{XG}}^2\delta\tau}. \tag{20}$$

The noise on the cross-correlation function in scenario D is

$$\sigma = (T\delta\tau)^{-1/2}\left(\eta\Phi N\left(\eta_{\mathrm{YX}} + \eta_{\mathrm{XG}}\right)/2 + D\right)^{-1} \tag{21}$$

and the threshold for the bunching amplitude to be higher than the noise is

$$T > \frac{16(\eta\Phi N(\eta_{\mathrm{YX}} + \eta_{\mathrm{XG}})/2 + D)^2}{\eta^4\eta_{\mathrm{YX}}^2\eta_{\mathrm{XG}}^2\Phi^2N^2k_{\mathrm{XG}}^2\delta\tau}. \tag{22}$$

SUPPLEMENTARY NOTE 4: NECESSITY OF TWO INDEPENDENT DETECTORS

To prove the occurrence of photon cutting in Pr^{3+}, we use an experiment in which we spectrally split the cascade emission over two separate detectors (see Fig. 2b in the main text). Photon bunching is observed as an increased likelihood of detecting a photon at detector 2 after detection of a photon at detector 1 in the cross-correlation function. Photon bunching is also visible in the experiment without spectral separation (grey in Fig. 3d in the main text), where photons are divided randomly over two detectors using a 50/50 beamsplitter. In principle, it would be possible to detect all photons with one detector and calculate the second-order auto-correlation function of the photon detection trace. However, detector dead-time and afterpulsing make an accurate experiment with one detector impossible. To illustrate this, Supplementary Fig. 4 shows the four possible correlations that can be calculated from the data collected by the two detectors in an experiment with spectral separation. The data used this figure are the same as those of Fig. 2f in the main text.

Cross-correlating the signals of the two detectors (Supplementary Fig. 4a,b) generates the cross-correlation function as plotted it in Fig. 2f in the main text. After a blue photon on detector 1 the intensity of red-green emission on detector 2 is increased temporarily (Supplementary Fig. 4a), but not the other way around (Supplementary Fig. 4b). This is the photon-bunching effect due to cascade emission, that we discuss and analyse in detail in the main text. Auto-correlating the separate detector signals (Supplementary Fig. 4c,d) shows bunching, too. This is unexpected, because it would imply that a blue photon is followed by a second blue photon (Supplementary Fig. 4c), or a red-green photon by a second red-green photon (Supplementary Fig. 4d). This is not what happens in Pr^{3+}. The bunching signal in the auto-correlation functions is due to detector afterpulsing, that is a false second detector click quickly after the detection and registration of an actual photon.[8, 9] This behaviour is observed to some extent for all available photomultiplier tubes and avalanche photodiodes. To avoid experimental artefacts, photon bunching experiments should therefore be performed in a two-detector setup, even if spectral separation is not possible.

SUPPLEMENTARY REFERENCES

[1] Herden, B., Meijerink, A., Rabouw, F. T., Haase, M. & Jüstel, T. On the efficient luminescence of β-Na($La_{1-x}Pr_x$)F_4. *J. Lumin.* **146**, 302–306 (2014).

[2] Wilson, M. W. B. *et al.* Ultrafast Dynamics of Exciton Fission in Polycrystalline Pentacene. *J. Am. Chem. Soc.* **133**, 11830–11833 (2011).

[3] Tabachnyk, M. *et al.* Resonant energy transfer of triplet excitons from pentacene to PbSe nanocrystals. *Nat. Mater.* **13**, 1033–1038 (2014).

[4] Thompson, N. J. *et al.* Energy harvesting of non-emissive triplet excitons in tetracene by emissive PbS nanocrystals. *Nat. Mater.* **13**, 1039–1043 (2014).

[5] Timmerman, D., Valenta, J., Dohnalová, K., de Boer, W. D. A. M. & Gregorkiewicz, T. Step-like enhancement of luminescence quantum yield of silicon nanocrystals. *Nat. Nanotechnol.* **6**, 710–713 (2011).

[6] Vergeer, P. *et al.* Quantum cutting by cooperative energy transfer in $Yb_xY_{1-x}PO_4$: Tb^{3+}. *Phys. Rev. B* **71**, 014119 (2005).

[7] Bajzer, Z., Therneau, T. M., Sharp, J. C. & Prendergast, F. G. Maximum likelihood method for the analysis of time-resolved fluorescence decay curves. *Eur. Biophys. J.* **20**, 247–262 (1991).

[8] Hamamatsu Photonics. *Photomultiplier tubes: basics and applications* (2007). URL `https://www.hamamatsu.com/resources/pdf/etd/PMT_handbook_v3aE.pdf`.

[9] Yen, H. T., Lin, S. D. & Tsai, C. M. A simple method to characterize the afterpulsing effect in single photon avalanche photodiode. *J. Appl. Phys.* **104**, 054504 (2008).
